# kinCSM: Using graph‐based signatures to predict small molecule CDK2 inhibitors

**DOI:** 10.1002/pro.4453

**Published:** 2022-10-26

**Authors:** Yunzhuo Zhou, Raghad Al‐Jarf, Azadeh Alavi, Thanh Binh Nguyen, Carlos H. M. Rodrigues, Douglas E. V. Pires, David B. Ascher

**Affiliations:** ^1^ School of Chemistry and Molecular Biosciences University of Queensland Brisbane Queensland Australia; ^2^ Structural Biology and Bioinformatics, Department of Biochemistry University of Melbourne Melbourne Victoria Australia; ^3^ Systems and Computational Biology, Bio21 Institute University of Melbourne Melbourne Victoria Australia; ^4^ Computational Biology and Clinical Informatics Baker Heart and Diabetes Institute Melbourne Victoria Australia; ^5^ School of Computing and Information Systems University of Melbourne Melbourne Victoria Australia

**Keywords:** bioactivity prediction, CDK2 inhibitors, graph‐based signatures, kinase inhibitors, machine learning, small molecules

## Abstract

Protein phosphorylation acts as an essential on/off switch in many cellular signaling pathways. This has led to ongoing interest in targeting kinases for therapeutic intervention. Computer‐aided drug discovery has been proven a useful and cost‐effective approach for facilitating prioritization and enrichment of screening libraries, but limited effort has been devoted providing insights on what makes a potent kinase inhibitor. To fill this gap, here we developed kinCSM, an integrative computational tool capable of accurately identifying potent cyclin‐dependent kinase 2 (CDK2) inhibitors, quantitatively predicting CDK2 ligand–kinase inhibition constants (pK_i_) and classifying different types of inhibitors based on their favorable binding modes. kinCSM predictive models were built using supervised learning and leveraged the concept of graph‐based signatures to capture both physicochemical properties and geometry properties of small molecules. CDK2 inhibitors were accurately identified with Matthew's Correlation Coefficients (MCC) of up to 0.74, and inhibition constants predicted with Pearson's correlation of up to 0.76, both with consistent performances of 0.66 and 0.68 on a nonredundant blind test, respectively. kinCSM was also able to identify the potential type of inhibition for a given molecule, achieving MCC of up to 0.80 on cross‐validation and 0.73 on the blind test. Analyzing the molecular composition of revealed enriched chemical fragments in CDK2 inhibitors and different types of inhibitors, which provides insights into the molecular mechanisms behind ligand–kinase interactions. kinCSM will be an invaluable tool to guide future kinase drug discovery. To aid the fast and accurate screening of CDK2 inhibitors, kinCSM is freely available at https://biosig.lab.uq.edu.au/kin_csm/.

## 1 INTRODUCTION

The human genome encodes more than 500 protein kinases, which catalyze the process of protein phosphorylation, where a phosphate group from ATP is transferred to the hydroxyl group of a serine/threonine/tyrosine residue in the target protein.^1^ Kinases are important in many cellular signaling processes, including cell growth, proliferation, apoptosis, and metabolism,^2^ with abnormal kinase regulation leading to a range of diseases, including cancer.^3^, ^4^, ^5^, ^6^ It has been proposed that over a third of human protein functions are regulated by phosphorylation, making kinases attractive targets for therapeutic interventions via inhibition or modulation.

Developing kinase inhibitors via the traditional drug development process, however, is a time‐consuming and costly endeavor. To date, 71 inhibitors have been approved by the U.S. Food and Drug Administration, targeting a small fraction of human kinases.^7^ While the traditional in vitro experiments for hit discovery are challenging and usually present low hit‐rates, data availability emerging from these efforts has led to developments in virtual screening, a time‐ and cost‐effective approach to enable improvement in discovery rates and prioritization of compounds.^8^ One approach that has successfully leveraged this data has been quantitative structure–activity relationship (QSAR)^9^ analyses, which have been playing an important role in drug discovery efforts.^10^ Balachandar et al. identified potent inhibitors targeting eight kinases by using deep learning models,^11^ and Govinda et al. predicted drug‐kinase inhibition constant (pK_i_) for a wide range of kinases.^12^ Additionally, Miljković et al. classified different types of inhibition based on binding modes by considering a ligand‐based approach.^13^ Although these models represent a significant contribution to the field, they presented poor performance and generalization capabilities, and provided limited biological insight into what physicochemical properties are required for the design of new potent kinase inhibitors, for different favorable binding modes.

Cyclin‐dependent kinases (CDKs) within the family of Ser/Thr kinases can drive the cell cycle propagation upon bindings to cyclins. They have become popular chemotherapeutic targets for different types of cancers. While a number of studies have been focused on CDK4/6 inhibitors to mediate tumor cell cycle arrest, CDK2 can also be a promising target to overcome drug resistance to CDK4/6 inhibitors.^14^ To our knowledge, there has been no freely accessible tool dedicated to predict the potency of CDK2‐targeting small molecules and their favorable binding modes as an assembly.

**FIGURE 1:**
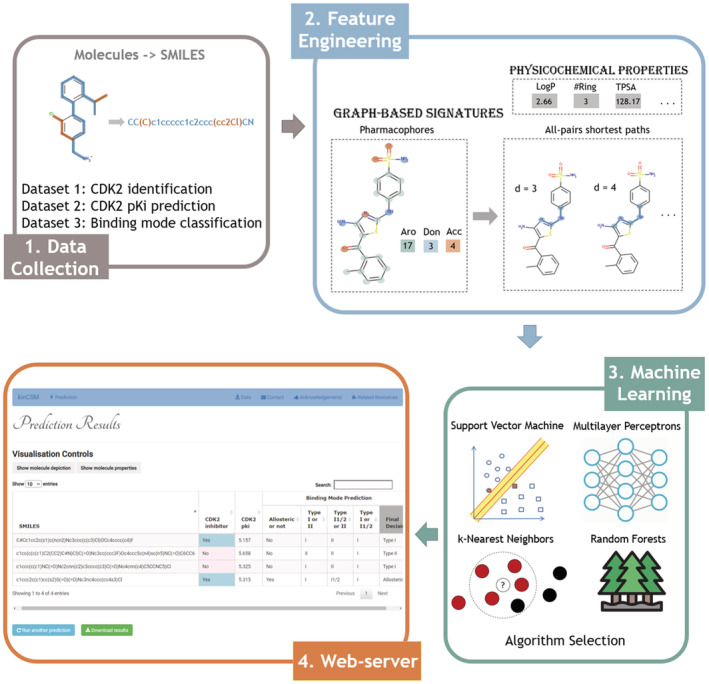
Methodology workflow. There were four steps involved in the methodology. First, molecules in SMILES representation and prediction labels were collected from three different sources for the three aims. After that, features were generated by pkCSM, including both physicochemical properties and graph‐based patterns. These features were input into different machine learning algorithms, trained using 10‐fold cross‐validation and tested on independent blind test sets. Finally, a freely available web server was developed

We have previously shown that the concept of graph‐based signatures could be used to model both protein and small molecule structures,^15^, ^16^, ^17^, ^18^, ^19^, ^20^ capturing both geometry and physicochemical properties.^21^, ^22^, ^23^, ^24^ Leveraging this concept, we developed kinCSM (Figure 1), a new predictive tool dedicated to identify potent CDK2 inhibitors. The method has three different predictive capabilities. First, it accurately identifies potential CDK2 inhibitors with IC50 <10 μM. Second, it quantitatively measures potency by predicting the inhibition constant (pK_i_), allowing compounds to be ranked and prioritized. Finally, it also enables the identification of the mode of inhibition. We show kinCSM performs as well as or better than similar methods and can generate biological insights into what makes potent CDK2 inhibitors.

## 2 RESULTS AND DISCUSSION

### 2.1 Associating molecular properties with CDK2 inhibition

By analyzing the general physicochemical properties of compounds, we found no strong correlation between independent molecular features and the inhibition constant, pK_i_ (Pearson's correlation coefficient of up to 0.21). Across our datasets, both CDK2 inhibitors (IC50 < 10 μM) and non‐inhibitors (IC50 ≥ 10 μM) generally followed Lipinski's rule of five (RO5)^25^ and Veber's Rule,^26^ reflecting an intrinsic bias in the screening libraries routinely used. Most of the active molecules evaluated had no more than 10 hydrogen bond acceptors, <5 hydrogen bond donors, octanol–water partition coefficient (log *p*) <5, no more than 10 rotatable bonds, and TPSA <140 Å^2^ (Figure S2).

Despite a modest correlation between inhibition strength and drug‐likeness properties, some physicochemical properties did distinguish between CDK2 inhibitors and non‐inhibitors. Potent CDK2 inhibitors had a lower log *p* (Figure S2(C); *p*‐value <0.001, using a two‐sample Kolmogorov–Smirnov test), indicating they are more hydrophilic and are more likely to be distributed in aqueous regions such as blood serum. Consistent with this observation, inhibitors also had a larger TPSA (Figure S2(E); *p*‐value <0.001) compared to non‐inhibitors, reflecting a potential to establish more interactions with CDK2.

### 2.2 Molecular substructure mining

**FIGURE 2:**
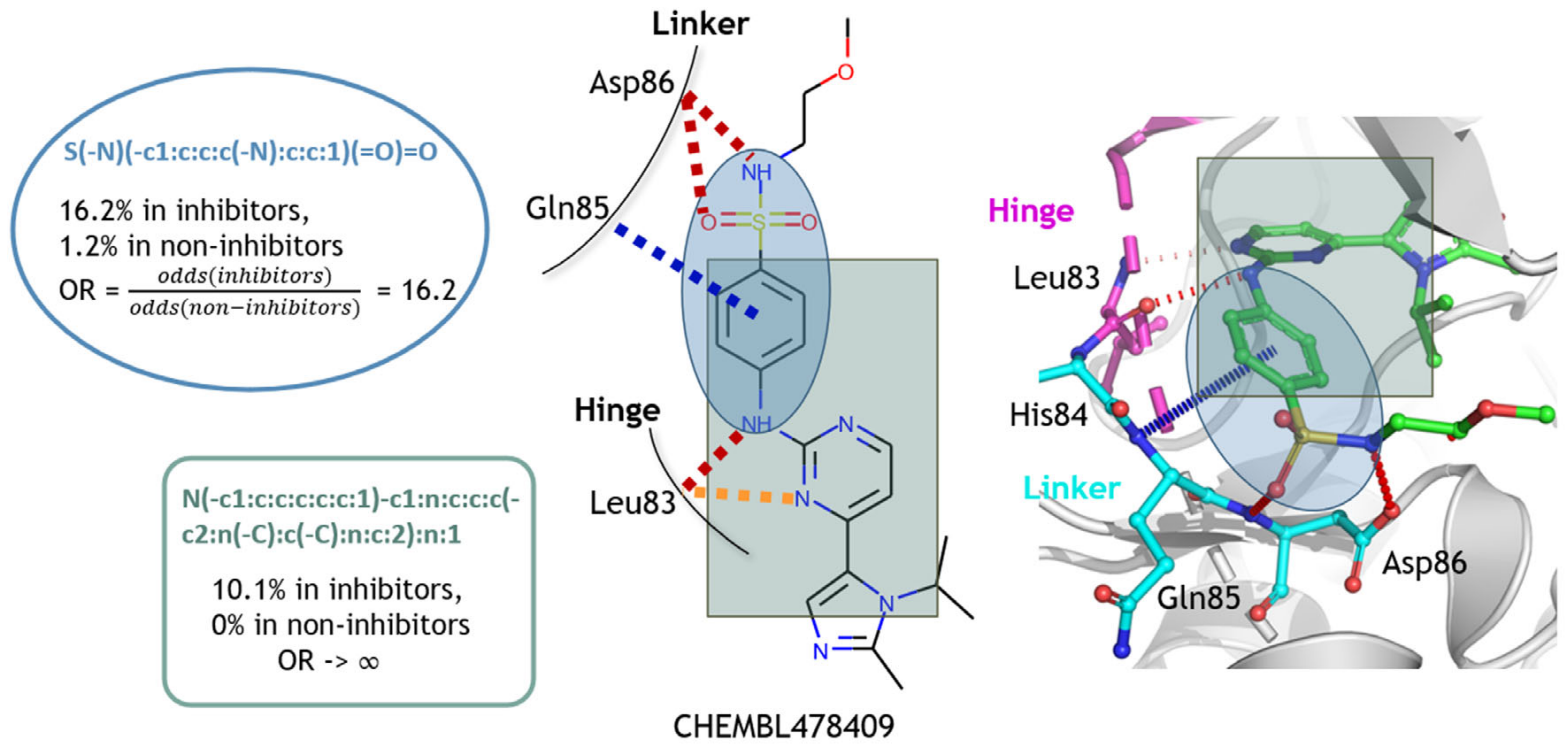
Substructure enrichment in CDK2 inhibitors (IC50 < 10 μM). The top left fragment (in blue), *sulfanilamide*, contains a sulfonamide, two hydrogen donors (nitrogen atoms at the top and the bottom) and one acceptor (the oxygen atom). It occurs 16.2% in CDK2 inhibitors (1.6% in a general kinase inhibitor dataset^28^ against 420 human kinases, 7.5% in other CDKs among the same dataset), 1.2% in CDK2 non‐inhibitors, with an odds ratio of 16.2. The bottom right fragment, 2‐(*N*‐*Anilino*)*pyrimidine*, contains heterocyclic rings that can mimic the adenine part of the ATP. It occurs 10.1% in CDK2 inhibitors (0.06% in the general kinase inhibitor dataset, 0.91% in other CDKs), never in non‐inhibitors, so the odds ratio would tend to infinity. The two fragments appeared together 2% in CDK2 inhibitors (0.02% in the general kinase inhibitor dataset, 0.35% in other CDKs). The intermolecular interactions between the inhibitor (CHEMBL ID: 478409; PDB Chemical ID: FRT) and the CDK2 (PDB code: 2w05) are calculated using Arpeggio,^39^ where hydrogen bonds, donor‐π, and polar interactions are shown in red, blue, and yellow dashes, respectively

To further our understanding of the chemical landscape of known CDK2 inhibitors, we used molecular substructure mining to identify enriched chemical groups. Using the Molecular Substructure Miner (MoSS),^27^ we found two chemical fragments, *sulfanilamide* (16.2% support) and 2‐(*N‐Anilino*)*pyrimidine* (10.1% support) that occurred more frequently in CDK2 inhibitors compared to non‐inhibitors (Figure 2), and appeared together with 2% support. Moreover, to analyze whether these two fragments are selective for CDK2, we searched them against a library of more than 36,000 known multi‐kinase inhibitors for 420 human kinases (with pKi, pKd, or pIC50 ≥ 6),^28^ and found they occur much less frequently in other kinase inhibitors and even other types of CDK2 inhibitors (<1% support). Atoms in these enriched and selective groups include hydrogen bond donors and acceptors, which can form interactions with the linker and hinge region in CDK2. Additionally, the ring structures in the fragments can mimic the adenine component of ATP, which are important for competitive inhibitors.

Few studies have been devoted to exclusively annotate different types of available CDK2 inhibitors. While the limited binding mode information on CDK2 inhibitors does not allow us to search for enriched fragments and build a dedicated model on classifying different types of CDK2 inhibitors, the consistent binding modes of the same molecule with different kinases enabled us to utilize the information from other kinase–ligand structures, which can also be applied to CDK2 inhibitors. The enriched substructure (24.2% support) in Type II inhibitors is composed of a 1‐Phenylurea connected to a ring (Figure S3). The odds ratio is 64.7 compared to Type I, and 41.6 compared to Type I1/2, indicating confident enrichment. Urea can form a hydrogen bond donor–acceptor pair with the αC‐helix and DFG motif, consistent with experimentally solved structures. The nitrogen atoms can establish hydrogen bonds with the glutamate side chain, which is conserved in αC‐helix, while the carbonyl group can establish a hydrogen bond with the backbone amide of the aspartate in the DFG‐motif. The benzene ring close to the donor nitrogen can form aromatic interactions with the gatekeeper residue in the kinase, and a hydrophobic moiety (at the top right corner in Figure S3 shaded in blue) accommodates into the back pocket. Accordingly, the urea acts as a bridge between the two ring structures, extending the molecules into the gatekeeper and back pockets exposed by the DFG out and αC‐helix out kinase conformation.

Substructure enrichment for Type I, I1/2, and allosteric inhibitors was not thoroughly analyzed. Type I and I1/2 inhibitors form stronger interactions with the hinge region similar to ATP, without having access to the back pocket. As all of Type I, I1/2, and Type II inhibitors share common substructures capable of occupying the ATP binding site, no substructure was found exclusively in Type I and I1/2 inhibitors. Additionally, the limited sample size for allosteric inhibitors (32 in 10‐fold cross‐validation, 15 in blind test) did not allow for unbiased enrichment analysis.

### 2.3 Identifying CDK2 inhibitors

**FIGURE 3:**
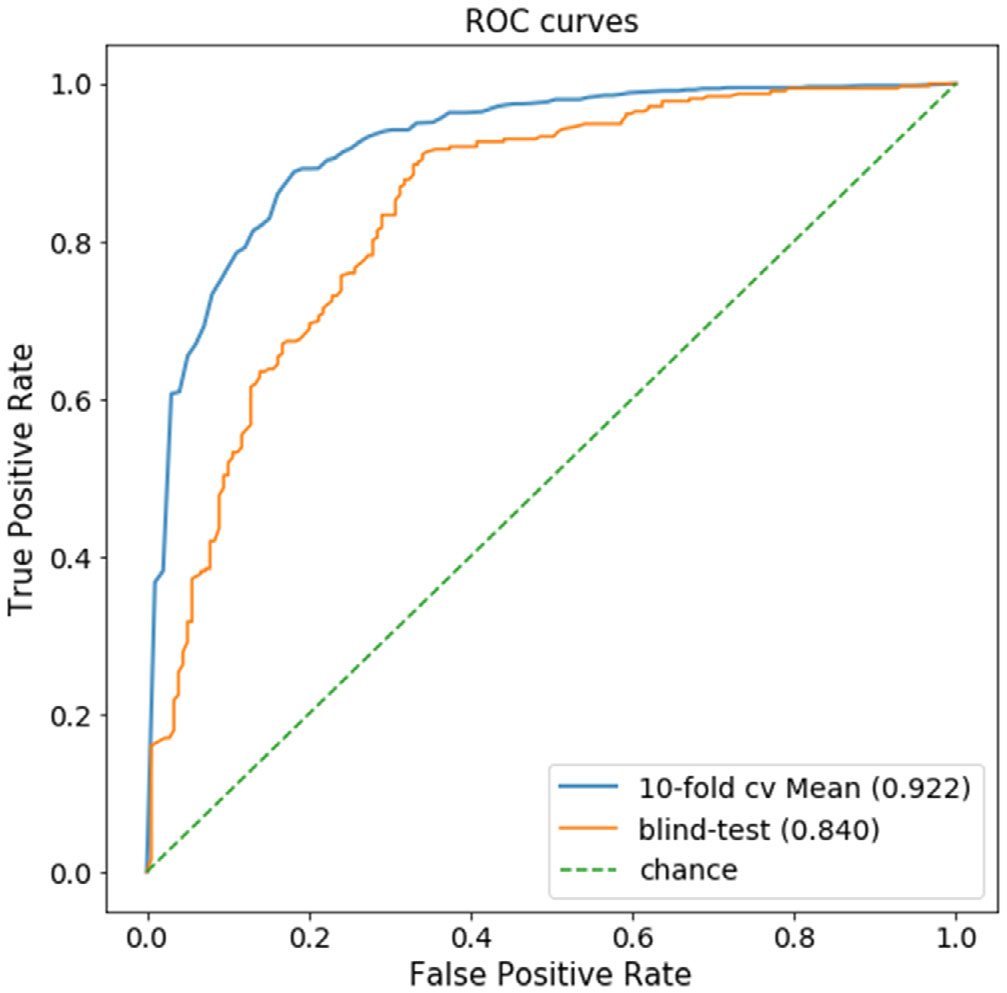
ROC curves for CDK2 inhibitor identification. Our model was able to correctly identify CDK2 inhibitors with AUC > 0.8 for both training and blind test sets. Here, we plot the mean ROC (with AUC 0.92) of all of the 10 folds instead of the overall ROC (with AUC 0.86) on training

Our predictive model was trained using different supervised learning algorithms. The best performing algorithm, Extra Tree Classifier (M5P) with 23 features (identified via feature selection), was chosen. Table 1 shows the overall model performance. Although the dataset used is relatively unbalanced (595 non‐inhibitors, 1040 inhibitors, using the cut‐off IC50 = 10 μM), the model still achieved high and consistent Matthew's Correlation Coefficients (MCCs) on both 10‐fold cross‐validation (0.74) and independent blind test set (0.66). F1 score (0.91 on cross‐validation and 0.88 on blind test) and AUC (0.86 on cross‐validation and 0.84 on blind test) also demonstrated model robustness (Figure 3). The performance metrics obtained via rigorous internal and external validation suggest potent CDK2 inhibitors can be correctly identified.

**TABLE 1:** Extra tree classifier performance for CDK2 inhibitor identification on training and blind test sets

	MCC	F1	AUC
10‐fold CV	0.74	0.91	0.86
Blind test	0.66	0.88	0.84

To shed light on properties that can explain differences between CDK2 inhibitors and non‐inhibitors, we conducted a two‐sample Kolmogorov–Smirnov test on the feature set. Figure S4(A) shows the top three features with the smallest *p*‐values. Inhibitors tend to have higher partial charges and van der Waals surface area contributions (PEOE_VSA12 attribute), a higher frequency of *sulfonamides*, and more *hydrogen bond donors* (*p*‐values <0.001). These characteristics reveal different non‐covalent interactions between enriched substructures (*sulfanilamide* and 2‐(*N‐Anilino)pyrimidine*) and CDK2, including electrostatic interactions, hydrogen bonds, and van der Waals forces, which can stabilize favorable inhibitor binding.

Compared to the deep learning models developed by Balachandar et al. on the same dataset, our classical machine learning algorithm has competitive performance. On the blind test, we achieved an AUC of 0.84, whereas Balachandar et al. achieved an AUC of 0.73.^11^ Although the performance results are not directly comparable since the training and test set splits are different, our model does demonstrate satisfactory generalization under a low‐redundant splitting strategy compared to the random split by Balachandar et al. The small score difference between the 10‐fold cross‐validation (0.86) and the blind test (0.84) provides further confidence in model robustness. Additionally, by investigating both the significant features and enriched substructures, we inferred discriminative physicochemical properties of potent inhibitors and discussed their biological significance. In contrast, no relevant biochemical insight was drawn from previous works,^11^ as features were encoded as bit strings to accommodate deep learning architectures, which are not explainable. Therefore, our model does not only have competitive prediction performance but also contributes to the detection of novel scaffolds among potent inhibitors and sheds light on their potential mode of action.

### 2.4 Predicting CDK2 ligand–kinase inhibition constant (pK_i_ )

**FIGURE 4:**
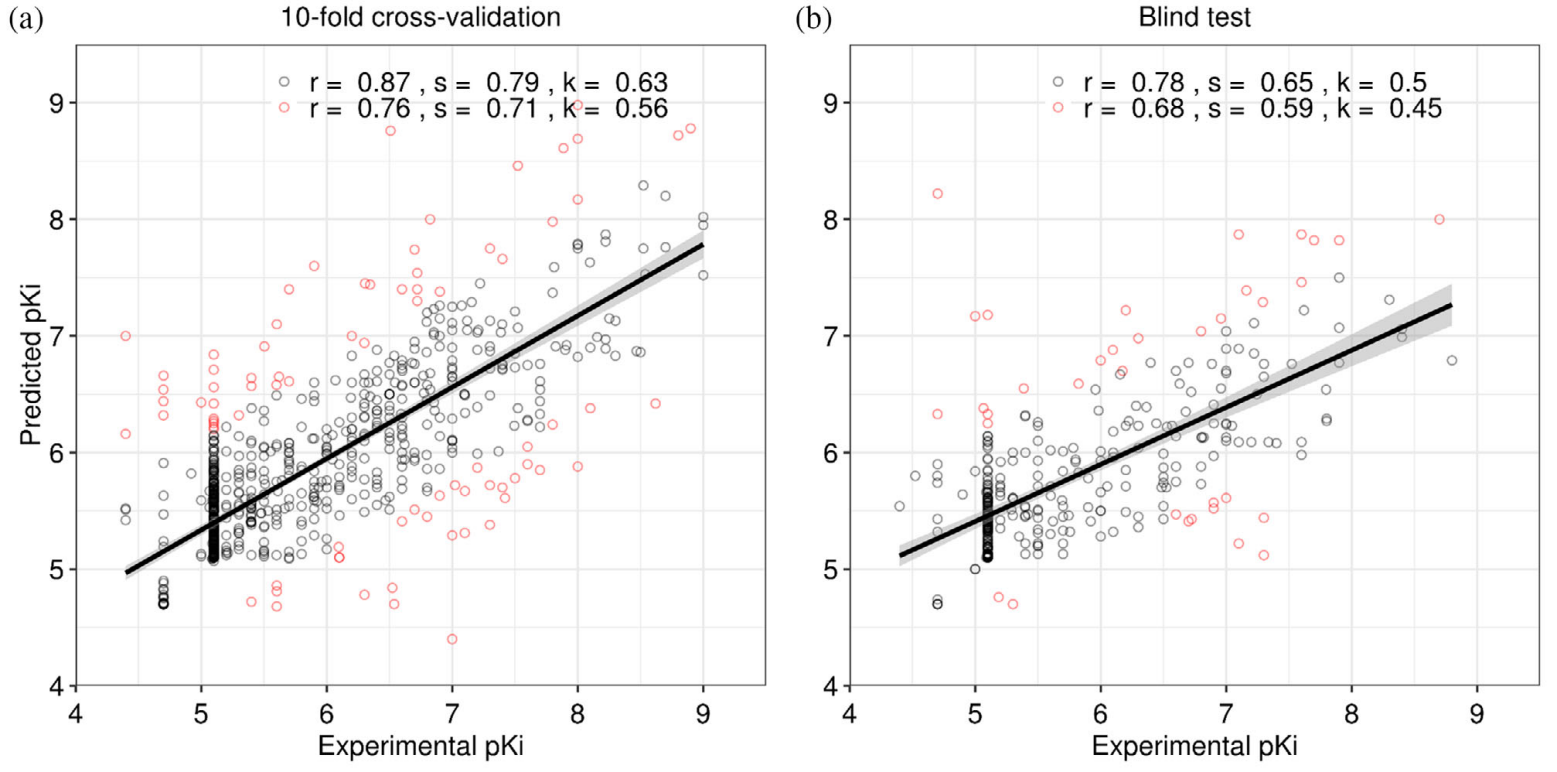
Regression plots for the 10‐fold cross‐validation and blind test sets on predicting pK_i_ . The plots depict the correlation between experimental and predicted pK_i_ . By removing the 10% outliers (highlighted in red), Pearson's correlation coefficients (*r*) increase from 0.76 to 0.87 on training, and from 0.68 to 0.78 on the blind test; Spearman's correlation coefficients (*s*) increase from 0.71 to 0.79 on training, and from 0.59 to 0.65 on the blind test; Kendall's correlation coefficients (*k*) increase from 0.56 to 0.63 on training, and from 0.45 to 0.50 on the blind test. Several molecules have qualified measurements (pK_i_ smaller than a given threshold) instead of precise measurements, leading to a concentration of points around pK_i_ of 5.1^40^

By predicting the pK_i_ values of small molecules, the inhibition strength can be quantified. A Random Forest Regressor with 22 features was trained and validated. Table 2 shows the overall model performance. We obtained a Pearson's correlation coefficient of 0.76 (RMSE of 0.62) on 10‐fold cross‐validation, and 0.68 (RMSE of 0.65) on an independent blind test set. The consistent performance between internal and external validation indicates model generalization. After removing 10% of outliers, Pearson's correlation coefficients increased to 0.87 on cross‐validation and 0.78 on the blind test (Figure 4). Here, no enriched substructures were observed exclusively in outlier molecules, indicating their structural diversity.

**TABLE 2:** Random Forest regressor performance on pK_i_ prediction

	Pearson	Spearman	Kendall	MSE	RMSE
10‐fold CV	0.76	0.71	0.56	0.39	0.62
Blind test	0.68	0.59	0.45	0.43	0.65

A recent study^29^ suggested an upper bound for scoring machine learning model performance on predicting drug‐kinase pK_i_, which has a Spearman's correlation of around 0.8 based on 10,000 samplings of replicated pK_i_ values. The performances obtained by kinCSM in predicting ligand‐CDK2 pK_i_ achieved Spearman's correlations of 0.71 on cross‐validation, and 0.59 on the blind test, approaching the maximal performance we would see using independent experimental measurements.

By dividing the molecules into two groups with the cut‐off pK_i_ value of 6, we were able to compare the physicochemical differences between the defined potent inhibitors (pK_i_ ≥ 6) and non‐inhibitors (pK_i_ < 6) in cell‐based assays using the two‐sample Kolmogorov–Smirnov test. Figure S4(B) depicts three significant features (*p*‐values <0.001) discriminating molecules with a high binding affinity using a more stringent threshold (pK_i_ ≥ 6). These features were consistent with those identified previously in our classifier, where a lower threshold was used (IC50 = 10 μM, that is, pIC50 = 5) as an initial crude screening. Moreover, the threshold pK_i_ = 6 also led to the highest performance when testing classification by regression (MCCs of 0.62 on cross validation, and 0.57 on blind‐test), highlighting the distinct differences between CDK2 inhibitors and non‐inhibitors under this threshold.

While the regression model with the pK_i_ cut‐off could also potentially be useful for classification purposes, in general, continuous labels can have higher variance compared to discrete classes, and may lead to poor classification performance. Instead, more information could be gained by considering the outputs from both classification and regression models. Accordingly, kinCSM provides a platform to quantify and rank inhibition strength in addition to inhibitor identification.

As a guideline to screen potent CDK2 inhibitors, we suggest users to take compounds, which meet the two following criterion (from crude to refined screening): (1) compounds identified as “inhibitor” by our classifier; (2) compounds with a moderately high pK_i_ ≥ 5.5 predicted by our regressor. Using the combined information, further validation results on active compounds and decoys (which are considered as non‐inhibitors that are challenging to classify) from DUD‐E^30^ CDK2 datasets are shown in Table S2, achieving an MCC of 0.52. Thus, the combined information from our classifier and regressor enabled a robust screening for CDK2 inhibitors.

### 2.5 Classifying different types of CDK2 inhibitors

The dataset for the classification of inhibitor types is highly unbalanced (1425 Type I, 394 Type I1/2, 190 Type II, and 47 allosteric inhibitors), which significantly increases the challenges of identifying the minority classes. However, our model was able to distinguish Type II inhibitors from Type I1/2 inhibitors, despite their smaller sample sizes. As shown in Table 3, the Type I1/2 versus Type II classifier achieved MCCs of 0.80 on cross‐validation and 0.73 on blind test sets. Additionally, it also achieved the highest AUC with 0.91 on the blind test set (Figure S5). The method has also identified allosteric inhibitors effectively, with an MCC of 0.68 on cross‐validation and 0.63 on the blind test.

**TABLE 3:** Performance of the inhibitor type classification model on training and blind test sets

Classifier	Metric	kinCSM cross validation	kinCSM blind test	Miljković et al.^13^ blind test
Type I vs. II	F1	0.73 (± 0.02)	0.64	0.71 (± 0.03)
	BACC	0.80 (± 0.01)	0.74	0.78 (± 0.02)
	MCC	0.73 (± 0.02)	0.65	0.70 (± 0.04)
Type I vs. I1/2	F1	0.54 (± 0.02)	0.43	0.58 (± 0.04)
	BACC	0.69 (± 0.01)	0.64	0.74 (± 0.02)
	MCC	0.50 (± 0.02)	0.41	0.47 (± 0.05)
Type I1/2 vs. II	F1	0.87 (± 0.01)	0.82	0.77 (± 0.03)
	BACC	0.90 (± 0.01)	0.88	0.82 (± 0.02)
	MCC	0.80 (± 0.02)	0.73	0.69 (± 0.03)
Allosteric or not	F1	0.64 (± 0.03)	0.57	0.36 (± 0.18)
	BACC	0.73 (± 0.02)	0.70	0.63 (± 0.07)
	MCC	0.68 (± 0.03)	0.63	0.48 (± 0.09)

Compared to the best machine learning model developed by Miljković et al.^13^ trained on 80% of the whole dataset under 10 different trials, and validated on a randomly generated external blind test set (20%), our model achieved higher MCCs in identifying allosteric inhibitors and distinguishing Type I1/2 and II inhibitors even when the blind test set (30%) presents low similarity with the training set (70%) and the model was trained on fewer data (Table 3). This means our model has a better generalization for unseen data when the sample size is limited and unbalanced.

Another challenge for this task was to do with the molecular structures of the three ATP competitive inhibitor types, which can be modeled as a continuum instead of distinct categories as the kinase conformation they bind changes in a stepwise manner.^31^ Type I inhibitors bind to the DFG‐in, αC‐helix in conformation, then the movement of αC‐helix (DFG‐in, αC‐helix out) allows binding of Type I1/2 inhibitors, and lastly, the DFG‐out, αC‐helix out conformation is recognized by Type II inhibitors. Two selected machine learning features (fluorine and hydrophobe counts) demonstrate this continuum (Figure S6(A,B)). The distributions of Type I1/2 inhibitors can be visualized as a mixture of Type I and Type II inhibitors, biased toward Type I. This may suggest that the shared substructures between types of favorable binding mode can affect model performance.

Being positioned in the middle of the continuum, Type I1/2 becomes the most challenging class, even though it has an adequate sample size. The Type I versus Type I1/2 classifier achieved the lowest performance (MCC of 0.50 on cross‐validation, and 0.41 on blind test, shown in Table 3). After integrating the prediction outcomes from the four binary classification models, a large proportion of Type I1/2 inhibitors were wrongly classified as Type I inhibitors (Figure S7). One possible reason is that Type I1/2 inhibitors share a larger proportion of common substructures with Type I inhibitors in comparison with Type II inhibitors. Although Type I1/2 inhibitors can form interactions with residues in the gatekeeper pocket, making them distinguishable from Type I, this characteristic may not be captured by our model. Rather, their strong affinity with the hinge region leads to similar physicochemical properties (e.g., low log *p* as shown in Figure S6(C)) as Type I inhibitors. Nevertheless, our model does capture features capable of distinguishing Type I1/2 inhibitors from others (e.g., higher frequency of nitrogen‐containing functional groups attached to aromatics, as shown in Figure S6(D)—Welch two‐sample *t*‐test *p*‐values <0.001 compared to Type I and II).

Although Type II inhibitors have a distinctive characteristic (back pocket access), a larger sample size still causes biased predictions toward Type I inhibitors (Figure S7). The Type I versus II classifier achieved MCC of 0.73 and 0.65 for 10‐fold cross‐validation and blind test, respectively (Table 3). However, insightful features were captured by our model. Type II inhibitors have higher log *p* (*p*‐values <0.001 compared to Type I and I1/2) as shown in Figure S6(C), which means they are more hydrophobic. This is caused by their special interactions with the kinase hydrophobic back pocket. Additionally, fluorine and urea occur more frequently in Type II inhibitors (*p*‐values <0.001, Figure S6(A,E)). This may suggest both of them can contribute to interactions with the back pocket.

### 2.6 kinCSM web server

**FIGURE 5:**
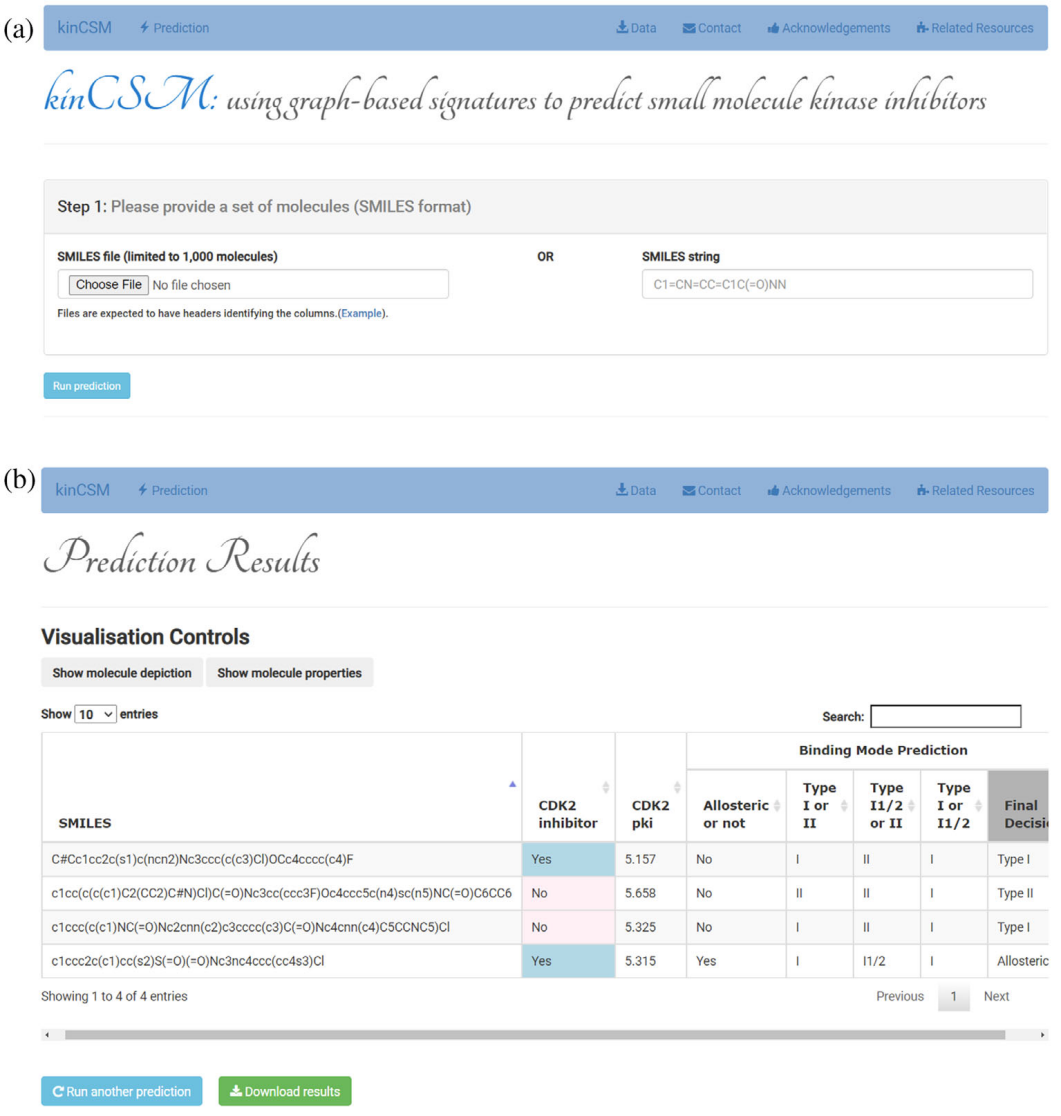
kinCSM Web server interface. (a) The submission page for kinCSM. Users can provide a molecule as a SMILES string, or upload a file containing multiple SMILES strings. (b) The results page for multiple molecule submission. Results are presented in a table, including predictions on CDK2 inhibitor (Yes or No), CDK2 pK_i_ , binding modes based on different binary classifiers, and final decisions. Users also have the choice to show molecule depiction and properties via visualization controls, as well as redirecting to toxicity and selectivity predictions by toxCSM ^32^ and SwissTarget, ^33^ respectively

kinCSM has been made freely available through an easy–to–use web interface at https://biosig.lab.uq.edu.au/kin_csm/. Users can identify CDK2 inhibitors, predict CDK2 pK_i_ and possible binding modes by providing a single molecule or a list of molecules as SMILES strings (Figure 5). Moreover, users can also predict the toxicity profiles via toxCSM,^32^ and selectivity profiles via SwissTarget^33^ by clicking on the links on the result page to further prioritize safer, less toxic, and more selective CDK2 inhibitors for clinical usage.

## 3 CONCLUSIONS

Here, we developed kinCSM, the first predictive tool to identify CDK2 inhibitors, predict CDK2 Ligand–Kinase Inhibition Constant (pK_i_), and classify different types of inhibitors in a single resource. This tool can be used to study both the binding affinity and favorable binding modes of CDK2 inhibitors.

Using the concept of graph‐based signatures, our model not only achieved high prediction performance but also inferred distinctive physicochemical properties that are supported by substructure mining. We have made the kinCSM web server freely available at https://biosig.lab.uq.edu.au/kin_csm/.

We anticipate further model optimization by generating substructure descriptors and oversampling the minor class in the future. The model can also be trained to target different kinases for inhibitor selectivity studies. This may create extra value for drug development. We believe kinCSM would be a useful tool for accelerating CDK2 inhibitor drug screening and improving hit rates.

## 4 MATERIALS AND METHODS

### 4.1 Datasets

Molecules with labels were curated from three different literature sources^11^, ^12^, ^13^ for the three aims, and converted into SMILES strings. The label distributions of the three datasets are all unbalanced to some extent. Dataset 1 has more CDK2 inhibitors (IC50 < 10 μM, 63.6%) than non‐inhibitors (IC50 ≥ 10 μM, 36.4%), without information on the exact IC50 values. The pK_i_ distribution in Dataset 2 has a peak at around 5. Additionally, most of the inhibitors discovered so far are Type I, and only a few allosteric inhibitors have been developed. This leads to the highly unbalanced dataset 3 (1425 Type I, 394 Type I1/2, 190 Type II, and 47 allosteric inhibitors) for inhibitor type classification. All datasets used in this study are available at https://biosig.lab.uq.edu.au/kin_csm/.

The training and test sets in the previous article were not low‐redundant, and will have led to data contamination and over‐estimated performance. To address this problem, in our work, the datasets were split into low‐redundancy training (70%) and blind test (30%). We ensured the molecules in the training and blind test sets have similar label distribution but are in different similarity clusters. The clusters were formed using the *rdkit.ML.Cluster.Butina* module in the cheminformatics toolkit RDKit^34^ according to the *TanimotoSimilarity*.^35^ The similarity thresholds (75% for Dataset 1 and 2, 55% for Dataset 3) were adjusted to ensure that around half of the molecules in the dataset are singletons, and the other half have at least one neighbor within their clusters.

### 4.2 Graph‐based signatures and feature selection

Molecular features for machine learning were extracted from SMILES strings as done previously.^21^, ^22^, ^23^ This approach has been successfully used on a variety of datasets to predict pharmacokinetic properties, including both classification (with categorical labels) and regression (with continuous labels). It generates both physicochemical features and graph‐based signatures, making it an effective way to represent molecules' properties.

The graph‐based signatures are distance patterns that are generated iteratively by the Cutoff Scanning Matrix (CSM) algorithm.^16^, ^17^, ^19^, ^36^ Molecules are modeled as a graph in an undirected and unweighted way, where atoms are represented as nodes, and bonds are represented as edges. Additionally, all atoms are labeled with pharmacophores (including *Acceptor*, *Donor*, *PosIonizable*, *NegIonizable*, *iAromatic*, and *Hydrophobe*) as shown in the bottom left panel of Figure S1. While scanning through the whole molecular graph, the distances between pharmacophore pairs are captured as a cumulative distribution using all‐pairs shortest paths (bottom right panel of Figure S1). This information can add extra values to the feature space, and therefore facilitate QSAR investigation.

### 4.3 Model selection and evaluation

Different machine learning models were trained and assessed under 10‐fold cross‐validation within the training set (70%). We then evaluated the trained models on the blind test set (30%) and compared the performance of the machine learning methods.

Specifically, in this study, we have compared the performance of the following popular machine learning techniques using the python Scikit‐learn library^37^: random forest (with 300 estimators), extra trees (with 300 estimators), multilayer perceptron (with the activation function “relu,” and the solver “adam”), and support vector machines (with the kernel “radial basis function”). Our evaluation result suggests that tree‐based methods lead to the highest performance for the regressor and most of the classifiers, except multilayer perceptron, which is the best method for Type I1/2 versus Type II classifiers.

Finally, the model performance was further evaluated by different metrics. MCC, F1 score and AUC for classification, Pearson's correlation coefficient (*r*), mean squared error (MSE), and root mean squared error (RMSE) for regression.

A bottom‐up greedy feature selection method was used according to MCC for classification, and Pearson's Correlation Coefficient (*r*) for regression, to simplify models and reduce noise.

### 4.4 Substructure mining

The SMILES strings were input into the MoSS^27^ to investigate substructure enrichment. We searched enriched substructures in a focused group of molecules (inhibitors) compared to a complementary set (non‐inhibitors). Discriminative fragments were found in CDK2 inhibitors compared to non‐inhibitors, and also for different types of CDK2 inhibitors in a pair‐wise manner. These substructures and patterns can further validate the features learned by our models, and also improve their overall interpretability. Finally, we studied the kinase–ligand interaction patterns by searching molecules enriched with these substructures in the Protein Data Bank (PDB)^38^.

The odds ratios for substructure enrichment were calculated based on the contingency tables obtained from control studies. They can quantify the association between enriched fragments and the inhibitors. Table S1 shows an example of the contingency table for the top left fragment (in the blue box) in Figure 2. The odds ratio was calculated as:

(1) $$\text{OR}=\frac{\text{odds}\left(\text{inhibitors}\right)}{\text{odds}\left(\text{non‐inhibitors}\right)}\;=\frac{168/872}{7/587}\approx 16.2$$

Odds ratios greater than one for both of the fragments demonstrate their confident enrichments in inhibitors.

### 4.5 Web server development

The web server front end was developed using Bootstrap framework version 3.3.7, and the back end was based on Python 2.7 via the Flask framework version 0.12.3 on a Linux server running Apache.

## FUNDING

This work was supported in part by a PhD scholarship from The Kingdom of Saudi Arabia (R.A); The National Health and Medical Research Council of Australia (GNT1174405 to D.B.A.), and The Victorian Government's Operational Infrastructure Support Program.

## AUTHOR CONTRIBUTIONS

**Yunzhuo Zhou:** Data curation (lead); formal analysis (lead); methodology (lead); writing (lead). **Raghad Al‐Jarf:** Data curation (supporting); methodology (supporting). **Azadeh Alavi:** Formal analysis (supporting). **Thanh Binh Nguyen:** Methodology (supporting); supervision (supporting). **Carlos H. M. Rodrigues:** Software (lead). **Douglas E. V. Pires**: Formal analysis (supporting); supervision (supporting). **David B. Ascher:** Conceptualization (lead); formal analysis (supporting); funding acquisition (lead); investigation (supporting); methodology (supporting); project administration (lead); software (supporting); supervision (lead); writing – review and editing (lead).

## CONFLICT OF INTEREST

The author declares that there is no conflict of interest that could be perceived as prejudicing the impartiality of the research reported.

## Supporting information


**Table S1.** Contingency table for a fragment (the blue fragment in Figure S2) enriched in CDK2 inhibitors.
**Table S2.** Model validation on a random subset of DUD‐E dataset based on the combined predictions from our classifier and regressor. The predicted inhibitors follow two conditions: (1) compounds identified as “inhibitor” by our classifier; (2) compounds with pK_i_ ≥ 5.5 predicted by our regressor (the threshold 5.5 was chosen because a concentration of molecules with experimental pK_i_ of 5.1 have predicted pK_i_ around 5.5 on the best fit line).
**Figure S1.** Features generated by pkCSM. Two types of features are generated, including molecular properties (top right panel) and distance distribution between pharmacophore pairs (bottom left and bottom right panels). The example molecule is a type I1/2 CDK2 inhibitor, named *RC‐3‐96* (PDB Chemical ID: 99Z).
**Figure S2.** Drug likeness property distributions for CDK2 inhibitors compared to non‐inhibitors. The violin plots show distributions of six physicochemical properties evaluating the drug likeness, including hydrogen acceptor and donor counts, log *p*, number of rotatable bonds, topological polar surface area (TPSA) and ring count. The white dot represents the median, and the range from the first quartile to the third quartile is shaded in black (applied to all the violin plots in this supplementary document). We found both inhibitors (*n* = 595, IC50 < 10 μM) and non‐inhibitors (n = 1,040, IC50 ≥ 10 μM) obey Lipinski's rule of five (RO5).
**Figure S3.** Substructure enrichment in type II inhibitors. Compound 61 (PDB Chemical ID: N61), a type II inhibitor of MAPK13. The enriched substructure (24.2% support, highlighted in red) contains a urea connected to a benzene ring on one side, and an undefined ring on the other side. The benzene ring forms a sulfur‐ π and hydrophobic interactions with the gatekeeper residue Met107, and a ring interaction with Phe169 in the DFG motif. Meanwhile, the oxygen in the urea forms a hydrogen bond with Asp168 in the DFG, and the two nitrogen atoms form hydrogen bonds with GLu72, a conserved residue in αC‐helix (PDB code: 4eyj).
**Figure S4.** Plots comparing CDK2 inhibitors and non‐inhibitors. (A) Inhibitors have higher *PEOE_VSA12* (which captures partial charges and van der Waals surface area contributions), a higher frequency of *sulfonamide* and hydrogen bond donors compared to non‐inhibitors (two‐sample Kolmogorov–Smirnov test *p*‐values <0.001). (B) Molecules with higher binding affinity (pK_i_ ≥ 6) are more likely to contain *Pyrrole*, they also have higher *fr_Ar_N* and *PEOE_VSA12* (two‐sample Kolmogorov–Smirnov test *p*‐values <0.001).
**Figure S5.** ROC curves for different binding mode classifiers. The ROC curves for type I versus II, type I versus I1/2, type I1/2 versus II and allosteric versus non‐allosteric are plotted. The type I1/2 versus II classifier has the highest Area Under the Curve, which means it can achieve higher performance by modifying the learned priors in our model.
**Figure S6.** Physicochemical properties of different types of kinase inhibitors. Panels A (*FCount*) and B (*Hydrophobe_Count*) show the structural continuum of type I, I1/2 and II inhibitors, where type II has the highest fluorine and hydrophobe pharmacophores (two‐sample Kolmogorov–Smirnov test *p*‐values <0.001 compared to both type I and I1/2). Type I1/2 shows a similar distribution of *MolLogP* with type I (panel C), but higher *fr_ArN* (panel D) with *p*‐values <0.001 makes it distinguishable from other types. Type II inhibitors have a higher frequency of urea (panel E) with *p*‐values <0.001. In the bar plots, the ranges from the first quartile to the third quartile are shown in black lines.
**Figure S7.** Integrated prediction performance on the blind test set. The prediction outcomes of binary classifiers were merged to enable multi‐class classification according to the majority vote.

## Data Availability

All dataset used to validate and test is freely available to download at: https://biosig.lab.uq.edu.au/kin_csm/data.
