# Supplementary material for: kinCSM: Using graph‐based signatures to predict small molecule CDK2 inhibitors
Source: Protein Sci. 2022 Oct 26;31(11):e4453. doi: 10.1002/pro.4453 (PMC9597374; doi:10.1002/pro.4453)
Supplement: Supplementary file 1 — Table S1. Contingency table for a fragment (the blue fragment in Figure S2) enriched in CDK2 inhibitors. Table S2. Model validation on a random subset of DUD‐E dataset based on the combined predictions from our classifier and regressor. The predicted inhibitors follow two conditions: (1) compounds identified as “inhibitor” by our classifier; (2) compounds with pKi ≥ 5.5 predicted by our regressor (the threshold 5.5 was chosen because a concentration of molecules with experimental pKi of 5.1 have predicted pKi around 5.5 on the best fit line). Figure S1. Features generated by pkCSM. Two types of features are generated, including molecular properties (top right panel) and distance distribution between pharmacophore pairs (bottom left and bottom right panels). The example molecule is a type I1/2 CDK2 inhibitor, named RC‐3‐96 (PDB Chemical ID: 99Z). Figure S2. Drug likeness property distributions for CDK2 inhibitors compared to non‐inhibitors. The violin plots show distributions of six physicochemical properties evaluating the drug likeness, including hydrogen acceptor and donor counts, log p, number of rotatable bonds, topological polar surface area (TPSA) and ring count. The white dot represents the median, and the range from the first quartile to the third quartile is shaded in black (applied to all the violin plots in this supplementary document). We found both inhibitors (n = 595, IC50 < 10 μM) and non‐inhibitors (n = 1,040, IC50 ≥ 10 μM) obey Lipinski's rule of five (RO5). Figure S3. Substructure enrichment in type II inhibitors. Compound 61 (PDB Chemical ID: N61), a type II inhibitor of MAPK13. The enriched substructure (24.2% support, highlighted in red) contains a urea connected to a benzene ring on one side, and an undefined ring on the other side. The benzene ring forms a sulfur‐ π and hydrophobic interactions with the gatekeeper residue Met107, and a ring interaction with Phe169 in the DFG motif. Meanwhile, the oxygen in the urea forms a hydrogen bond with A [file PRO-31-e4453-s001.docx]

**Supplementary Materials**

**kinCSM: using graph-based signatures to predict small molecule CDK2 kinase inhibitors**

Yunzhuo Zhou^1,2,3,4^, Raghad Al-Jarf^2,3,4^, Azadeh Alavi^2,3,4^, Thanh Binh Nguyen^1,2,3,4^ ,
Carlos H. M. Rodrigues^1,2,3,4^, Douglas E. V. Pires^1,2,3,4,5*^, David B. Ascher^1,2,3,4,*^

^1^ School of Chemistry and Molecular Biosciences, University of Queensland, Brisbane, Queensland, Australia

^2^Structural Biology and Bioinformatics, Department of Biochemistry, University of Melbourne, Melbourne, Victoria, Australia
^3^Systems and Computational Biology, Bio21 Institute, University of Melbourne, Melbourne, Victoria, Australia
^4^Computational Biology and Clinical Informatics, Baker Heart and Diabetes Institute, Melbourne, Victoria, Australia

^5^School of Computing and Information Systems, University of Melbourne, Melbourne, Victoria, Australia

*To whom correspondence should be addressed D.B.A. Tel: +61 90354794; Email: [d.ascher@uq.edu.au](mailto:d.ascher@uq.edu.au). Correspondence may also be addressed to D.E.V.P. [douglas.pires@unimelb.edu.au](about:blank).

**TABLES**

**Table S1.** Contingency table for a fragment (the blue fragment in Figure S2) enriched in CDK2 inhibitors.

|  | Inhibitors | Non-inhibitors |  |
| --- | --- | --- | --- |
| Cases (with the fragment) | 168 | 7 |  |
| Controls (without the fragment) | 872 | 587 |  |

**Table S2.** Model validation on a random subset of DUD-E dataset based on the combined predictions from our classifier and regressor. The predicted inhibitors follow two conditions: (1) compounds identified as ‘inhibitor’ by our classifier; (2) compounds with pK_i_ ≥ 5.5 predicted by our regressor (the threshold 5.5 was chosen because a concentration of molecules with experimental pK_i_ of 5.1 have predicted pK_i_ around 5.5 on the best fit line).

|  | Predicted Inhibitors | Predicted Decoys |  |
| --- | --- | --- | --- |
| Actual Inhibitors | 269 | 31 |  |
| Actual Decoys | 120 | 180 |  |

**FIGURES**

**
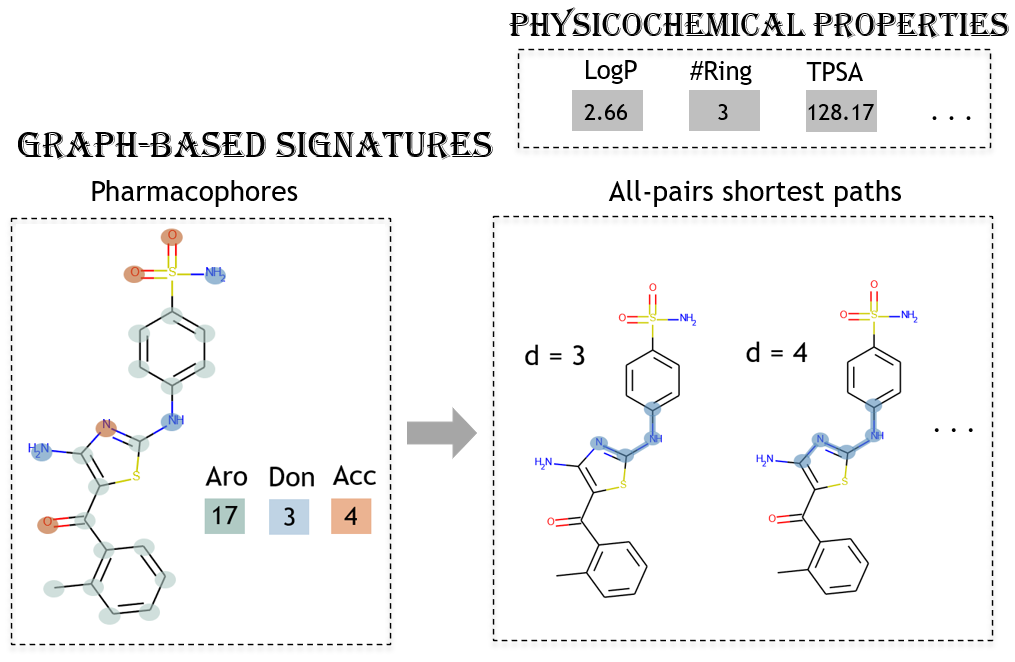
Figure S1. Features generated by pkCSM.** Two types of features are generated, including molecular properties (top right panel) and distance distribution between pharmacophore pairs (bottom left and bottom right panels). The example molecule is a type I1/2 CDK2 inhibitor, named *RC-3-96* (PDB Chemical ID: 99Z).


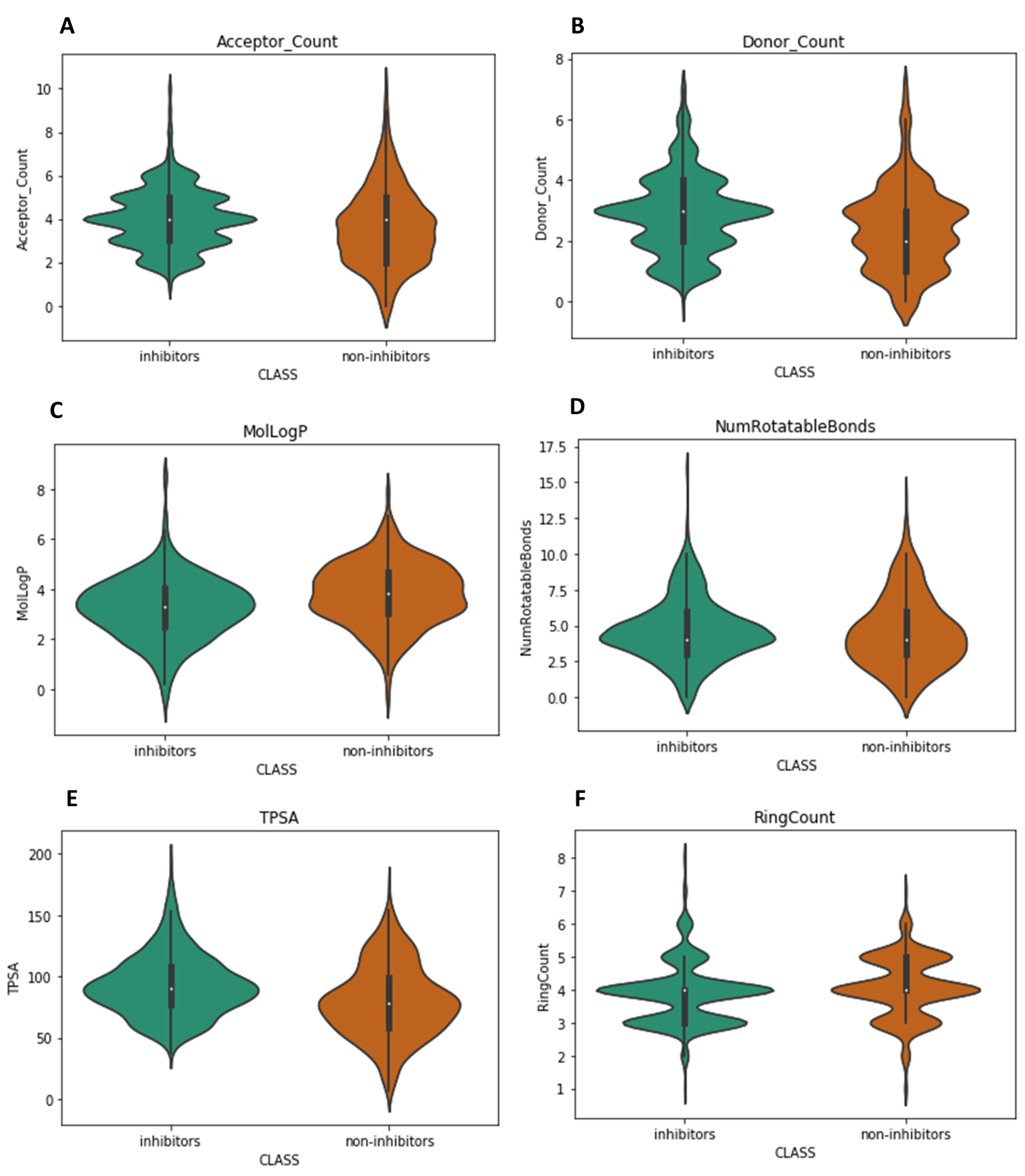


**Figure S2. Drug likeness property distributions for CDK2 inhibitors compared to non-inhibitors.** The violin plots show distributions of six physicochemical properties evaluating the drug likeness, including hydrogen acceptor and donor counts, log P, number of rotatable bonds, topological polar surface area (TPSA) and ring count. The white dot represents the median, and the range from the first quartile to the third quartile is shaded in black (applied to all the violin plots in this supplementary document). We found both inhibitors (n = 595, IC50 < 10 µM) and non-inhibitors (n = 1040, IC50 ≥ 10 µM) obey Lipinski's rule of five (RO5).


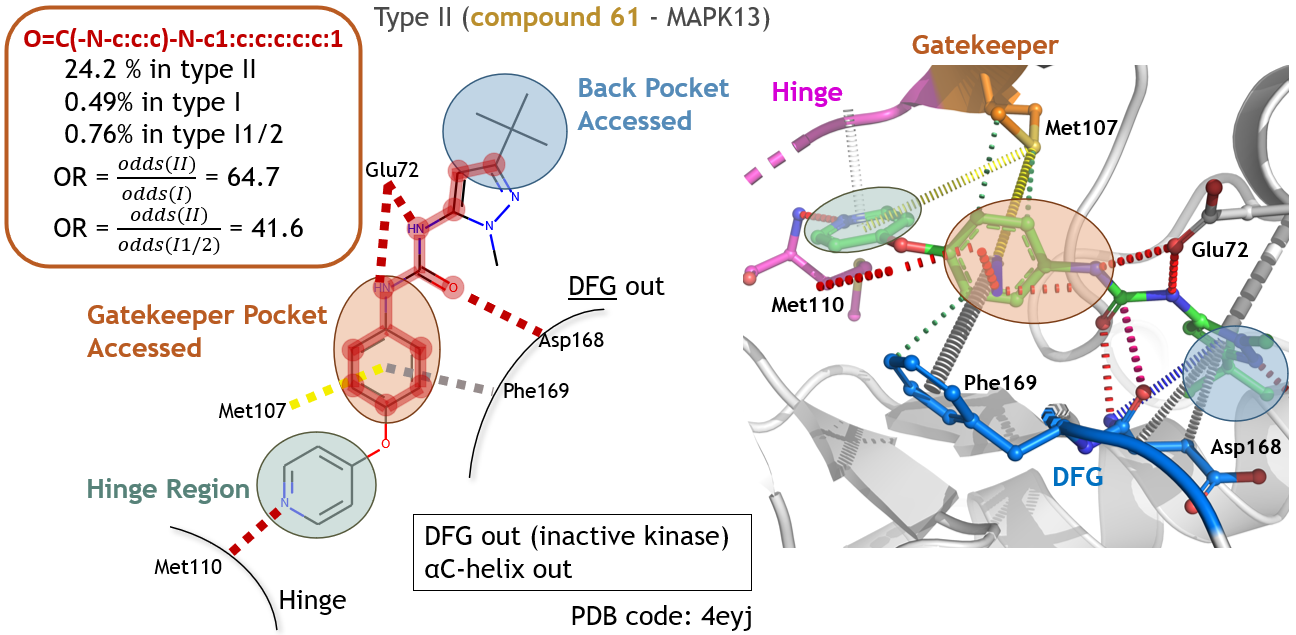


**Figure S3. Substructure enrichment in type II inhibitors.** Compound 61 (PDB Chemical ID: N61), a type II inhibitor of MAPK13. The enriched substructure (24.2% support, highlighted in red) contains a urea connected to a benzene ring on one side, and an undefined ring on the other side. The benzene ring forms a sulfur- π and hydrophobic interactions with the gatekeeper residue Met107, and a ring interaction with Phe169 in the DFG motif. Meanwhile, the oxygen in the urea forms a hydrogen bond with Asp168 in the DFG, and the two nitrogen atoms form hydrogen bonds with GLu72, a conserved residue in αC-helix (PDB code: 4eyj).

**
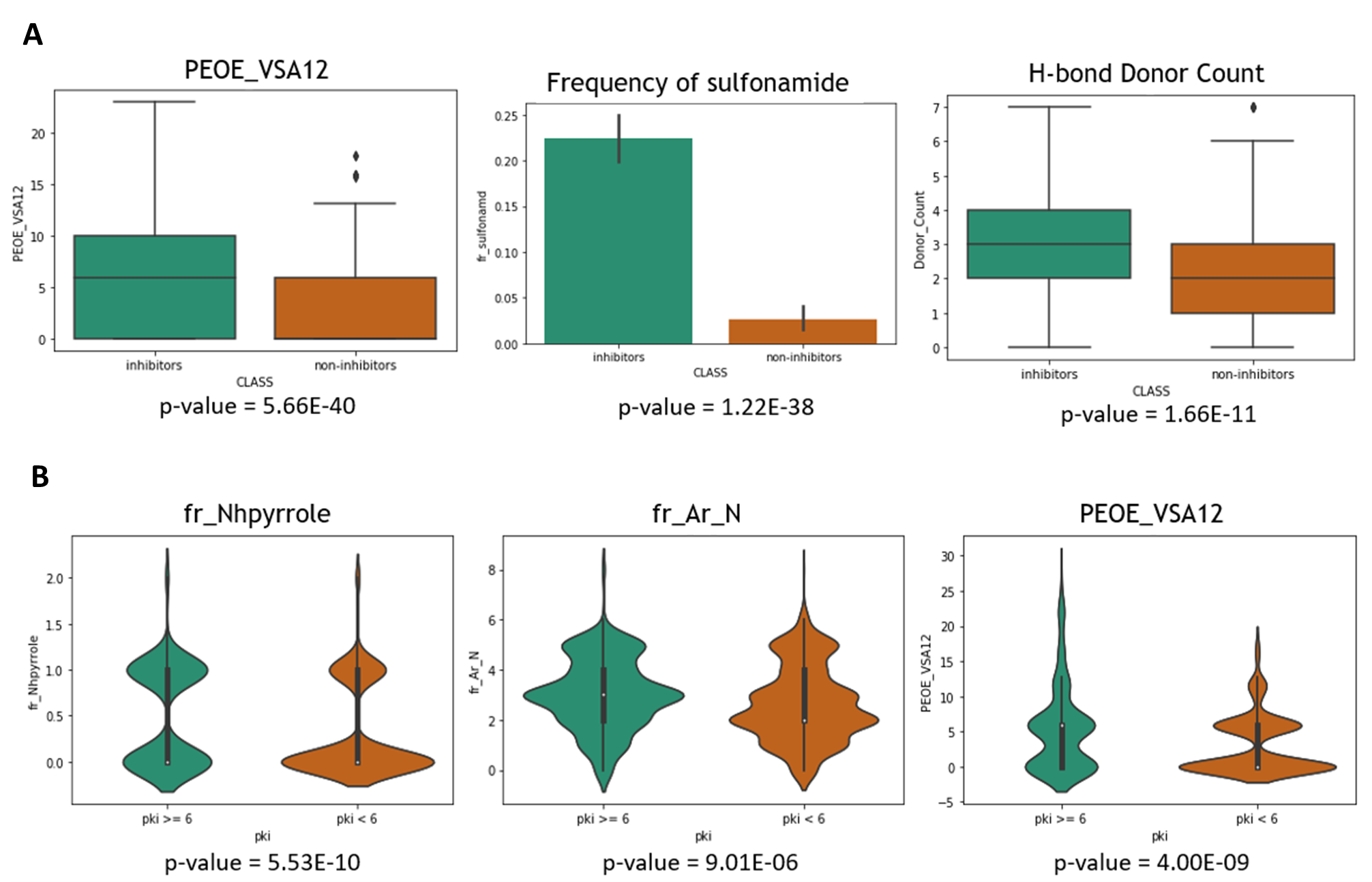
**

**Figure S4. Plots comparing CDK2 inhibitors and non-inhibitors.** **A)** Inhibitors have higher *PEOE_VSA12* (which captures partial charges and van der Waals surface area contributions), a higher frequency of *sulfonamide* and hydrogen bond donors compared to non-inhibitors (two-sample Kolmogorov-Smirnov test p-values < 0.001). **B)** Molecules with higher binding affinity (pK_i_ ≥ 6) are more likely to contain *Pyrrole*, they also have higher *fr_Ar_N* and *PEOE_VSA12* (two-sample Kolmogorov-Smirnov test p-values < 0.001).


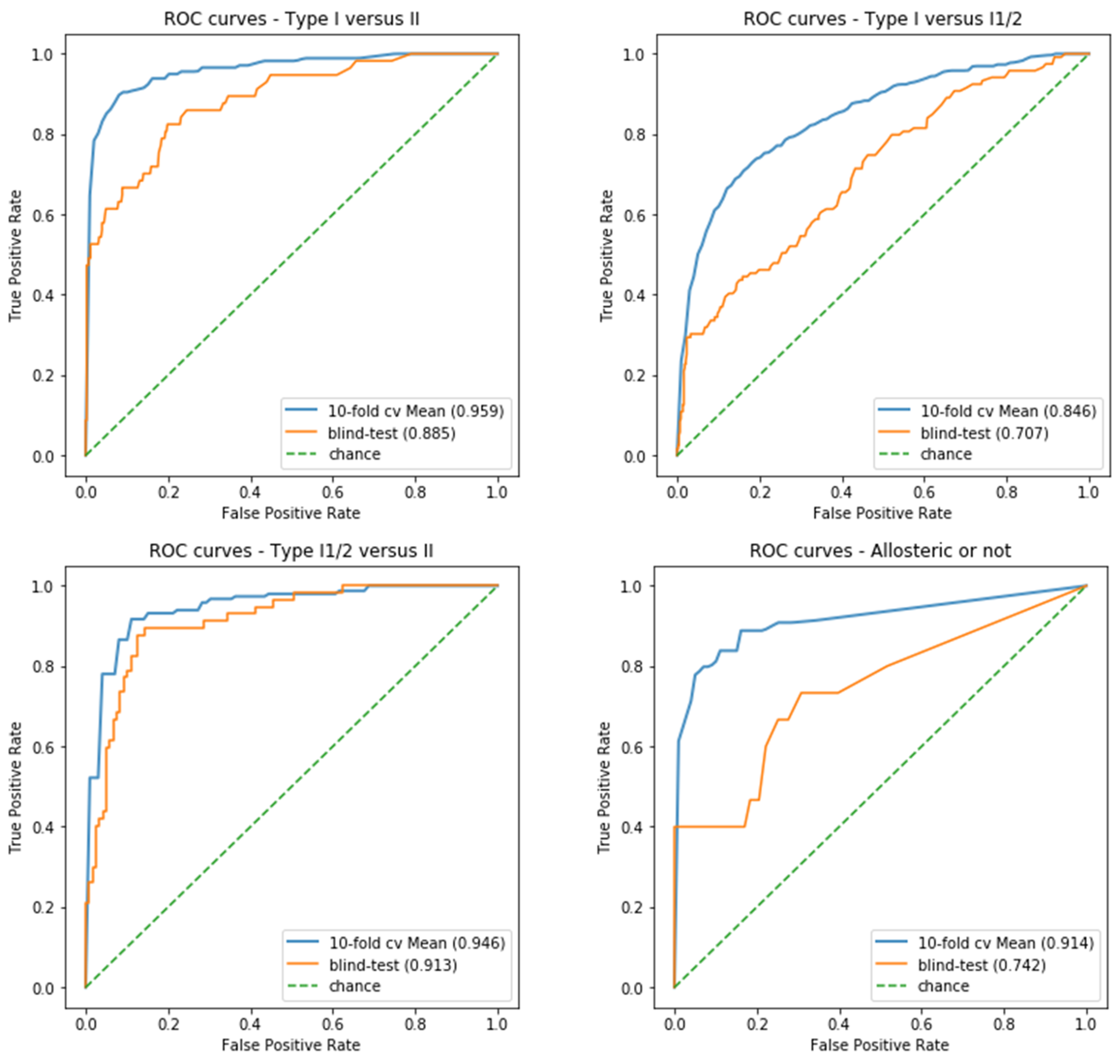


**Figure S5. ROC curves for different binding mode classifiers.** The ROC curves for type I versus II, type I versus I1/2, type I1/2 versus II and allosteric versus non-allosteric are plotted. The type I1/2 versus II classifier has the highest Area Under the Curve, which means it can achieve higher performance by modifying the learned priors in our model.


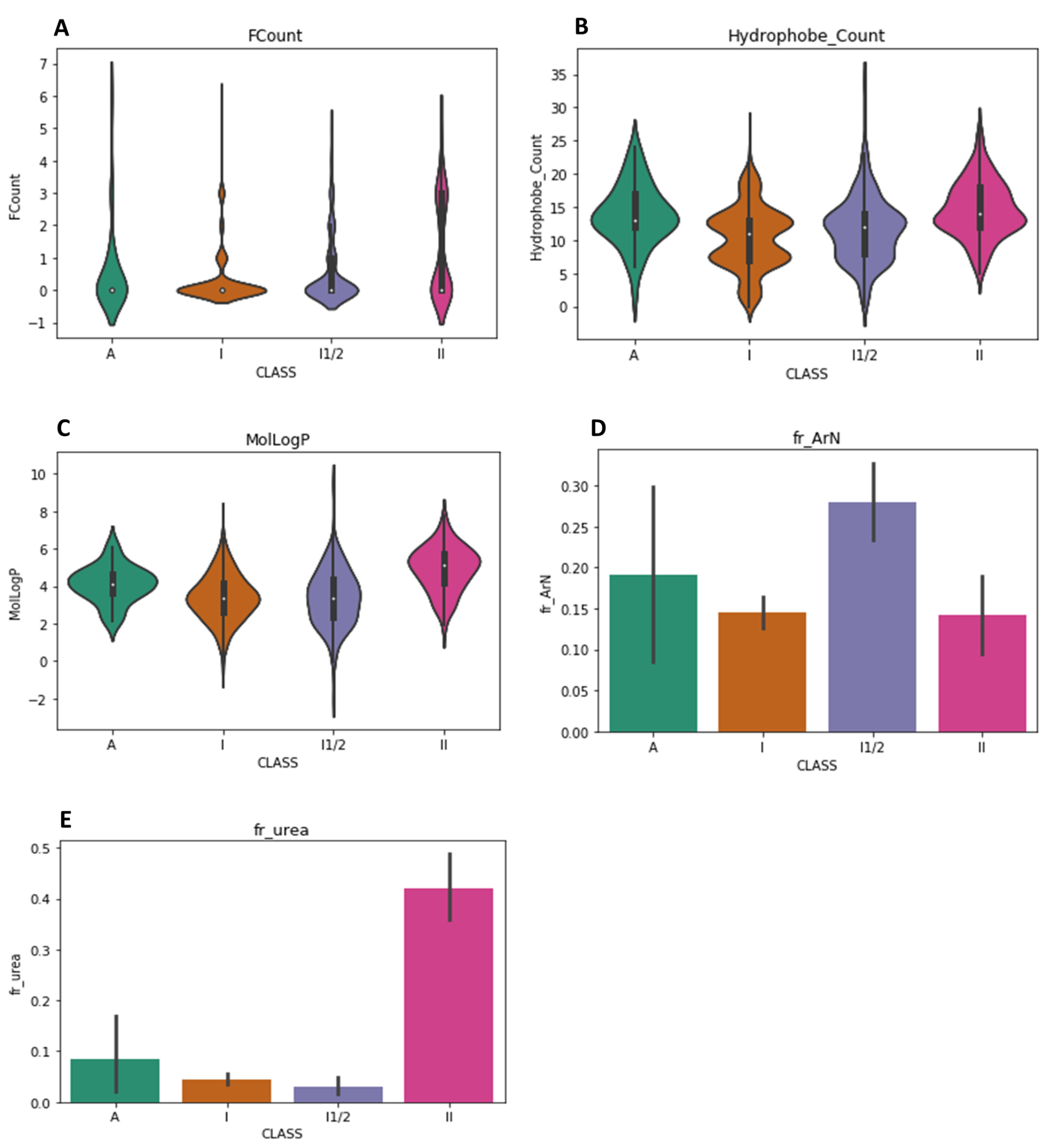


**Figure S6. Physicochemical properties of different types of kinase inhibitors.** Panels A (*FCount*) and B (*Hydrophobe_Count*) show the structural continuum of type I, I1/2 and II inhibitors, where type II has the highest fluorine and hydrophobe pharmacophores (two-sample Kolmogorov-Smirnov test p-values < 0.001 compared to both type I and I1/2). Type I1/2 shows a similar distribution of *MolLogP* with type I (panel C)*,* but higher *fr_ArN* (panel D) with p-values < 0.001 makes it distinguishable from other types. Type II inhibitors have a higher frequency of urea (panel E) with p-values < 0.001. In the bar plots, the ranges from the first quartile to the third quartile are shown in black lines.


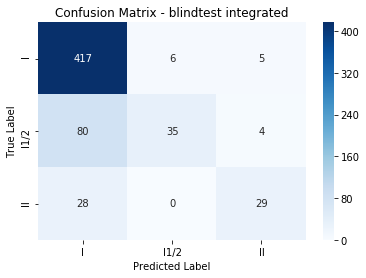


**Figure S7. Integrated prediction performance on the blind test set.** The prediction outcomes of binary classifiers were merged to enable multi-class classification according to the majority vote.
